# A randomized trial of Plasma-Lyte A and 0.9 % sodium chloride in acute pediatric gastroenteritis

**DOI:** 10.1186/s12887-016-0652-4

**Published:** 2016-08-02

**Authors:** Coburn H. Allen, Ran D. Goldman, Seema Bhatt, Harold K. Simon, Marc H. Gorelick, Philip R. Spandorfer, David M. Spiro, Sharon E. Mace, David W. Johnson, Eric A. Higginbotham, Hongyan Du, Brendan J. Smyth, Carol R. Schermer, Stuart L. Goldstein

**Affiliations:** 1 Department of Pediatrics, Dell Medical School at University of Texas at Austin, 4900 Mueller Blvd, Austin, TX 78746 USA; 2 Department of Pediatrics, British Columbia Children’s Hospital, University of British Columbia, Vancouver, BC Canada; 3 Department of Pediatrics, Cincinnati Children’s Hospital Medical Center, Cincinnati, OH USA; 4 Departments of Pediatrics and Emergency Medicine, Emory University/Children’s Healthcare of Atlanta, Atlanta, GA USA; 5 Pediatric Emergency Medicine, Children’s Hospital of Wisconsin, Milwaukee, WI USA; 6 Pediatric Emergency Medicine Associates, Children’s Healthcare of Atlanta, Atlanta, GA USA; 7 Pediatric Emergency Services, Oregon Health and Science University, Portland, OR USA; 8 Department of Emergency Medicine, Cleveland Clinic, Cleveland, OH USA; 9 Departments of Pediatrics, Pharmacology and Physiology, Alberta Children’s Hospital, Calgary, AB Canada; 10 Research and Development, Baxter Healthcare Corporation, Deerfield, IL USA; 11 Bristol-Myers Squibb, Pennington, NJ USA

**Keywords:** Balanced fluid therapy, Dehydration, Hyperchloremic metabolic acidosis, Plasma-Lyte A, Rehydration, Gastroenteritis

## Abstract

**Background:**

Compare the efficacy and safety of Plasma-Lyte A (PLA) versus 0.9 % sodium chloride (NaCl) intravenous (IV) fluid replacement in children with moderate to severe dehydration secondary to acute gastroenteritis (AGE).

**Methods:**

Prospective, randomized, double-blind study conducted at eight pediatric emergency departments (EDs) in the US and Canada (NCT#01234883). The primary outcome measure was serum bicarbonate level at 4 h. Secondary outcomes included safety and tolerability. The hypothesis was that PLA would be superior to 0.9 % NaCl in improvement of 4-h bicarbonate. Patients (*n* = 100) aged ≥6 months to <11 years with AGE-induced moderate-to-severe dehydration were enrolled. Patients with a baseline bicarbonate level ≤22 mEq/L formed the modified intent to treat (mITT) group.

**Results:**

At baseline, the treatment groups were comparable except that the PLA group was older. At hour 4, the PLA group had greater increases in serum bicarbonate from baseline than did the 0.9 % NaCl group (mean ± SD at 4 h: 18 ± 3.74 vs 18.0 ± 3.67; change from baseline of 1.6 and 0.0, respectively; *P* = .004). Both treatment groups received similar fluid volumes. The PLA group had less abdominal pain and better dehydration scores at hour 2 (both *P* = .03) but not at hour 4 (*P* = 0.15 and 0.08, respectively). No patient experienced clinically relevant worsening of laboratory findings or physical examination, and hospital admission rates were similar. One patient in each treatment group developed hyponatremia. Four patients developed hyperkalemia (PLA:1, 0.9 % NaCl:3).

**Conclusion:**

In comparison with 0.9 % NaCl, PLA for rehydration in children with AGE was well tolerated and led to more rapid improvement in serum bicarbonate and dehydration score.

**Trial registration:**

NCT#01234883 (Registration Date: November 3, 2010).

**Electronic supplementary material:**

The online version of this article (doi:10.1186/s12887-016-0652-4) contains supplementary material, which is available to authorized users.

## Background

Acute gastroenteritis (AGE) complicated by dehydration remains a major cause of childhood morbidity and mortality, requiring significant healthcare expenditure worldwide [1–3]. Approximately 179 million cases of AGE occur in the US each year [4–6]. Despite a decrease in positive laboratory diagnoses of AGE, likely attributable to rotavirus vaccination availability since 2006 [7], substantial disease remains. The fluid loss associated with AGE not only causes dehydration, but can lead to metabolic acidosis and electrolyte disturbances [1, 6, 8, 9].

Intravenous fluid therapy (IVT) is the mainstay of treatment for severe pediatric dehydration, and the requirement for continued IVT is the leading indication for hospitalization. However, recommendations for IVT are poorly standardized, and significant controversy exists as to the optimal fluid for use in children [10, 11]. In general, isotonic fluids are advised for acute rehydration with the most commonly administered fluids being 0.9 % sodium chloride (NaCl) and Lactated Ringer’s (LR) solution [12]. However, 0.9 % NaCl, which contains a supraphysiologic chloride concentration, can induce hyperchloremic metabolic acidosis (HCA), which can exacerbate the low serum bicarbonate levels often associated with diarrhea and poor perfusion from dehydration [13]. To prevent HCA, clinicians use LR, but concerns of hyponatremia due to low sodium concentration limit its use [12]. Thus, there is a substantial need to further evaluate alternative isotonic crystalloids as a treatment for AGE [10].

Plasma-Lyte A (PLA, Baxter Healthcare, Deerfield, IL), a balanced isotonic crystalloid, contains physiologic sodium, chloride, potassium, magnesium, and bicarbonate precursors in mEq/L: Na 140, K 5, Cl 98, Mg 3, Acetate 27, and gluconate 23, pH 7.4). It is utilized as a source of water and electrolytes or as an alkalinizing agent. Several studies in adults have reported a reduction in the incidence of hyperchloremia and metabolic acidosis with balanced solutions (eg, LR and PLA) over 0.9 % NaCl [14–16]. The osmolarity of Plasma-Lyte A is 294 mOsmol/L, within the pediatric reference range [17]. Of note, isotonic Plasma-Lyte has different naming conventions around the globe and hence different publications may refer to Plasmalyte A, Plasmalyte, or Plasmalyte 148. In the Unites States, there are 2 naming formulations which are identical in ionic composition but which differ in solution pH: Plasma-Lyte A (used here) which has a pH of 7.4 and Plasma-lyte 148 which has a pH of 5.5.

Plasma-Lyte A has not previously been studied specifically in the pediatric population. In addition to the physiologic chloride level, PLA contains acetate and gluconate, which serve as buffering agents. It may be preferred for children with AGE because it replaces water and electrolytes lost due to diarrhea and vomiting as well as bicarbonate lost in stool. PLA has the potential to reverse the acidosis that may contribute to the physical symptoms of nausea, vomiting, diarrhea, and abdominal pain [18, 19].

The hypothesis of this study was that PLA would be superior to 0.9 % NaCl in improvement of 4-h bicarbonate level and result in faster resolution of clinical signs and symptoms in children with AGE and dehydration.

## Methods

This prospective, randomized, triple-blind, company-sponsored, active-controlled study was conducted at 8 pediatric emergency departments (ED) in the US and Canada (NCT#01234883). Institutional ethics approval was obtained from each institution (Additional file 1: Table S1), and written informed consent was obtained from the parent/legal guardian of all children before randomization. Safety data were periodically monitored by an independent consultant (pediatric nephrologist, SLG) who was not involved in patient recruitment or management.

Patients ≥6 months to <11 years of age were eligible for enrollment if they presented to the ED with moderate-to-severe dehydration due to AGE, defined as ≥3 episodes of diarrhea or nonbilious vomiting within the previous 24 h and a Gorelick dehydration score ≥4 [20]. Screened patients had blood drawn for serum chemistry. A prerandomization fluid bolus of ≤20 mL/kg in the 4 h prior to enrollment was permitted. Exclusion criteria included AGE that did not require IVT per clinicians’ judgment, chronic health conditions such as renal failure affecting the ability to tolerate fluids or those that result in electrolyte abnormalities, or the use of prohibited medications (eg, antacids/anti-diarrhea or systemic corticosteroids within 24 or 72 h prior to presentation, respectively).

During the prestudy period, all patients received routine care, and oral rehydration therapy and IVT per clinician judgment. Screening included review of inclusion/exclusion criteria, complete medical history and physical exam, Gorelick score, abdominal pain assessment, and assessment of volume and type of prestudy IVT.

Concealed treatment allocation was via an Interactive Voice Recognition System/Interactive Web-based System. Eligible patients were randomly assigned in a 1:1 ratio to receive concealed bolus therapy with PLA (Multiple Electrolyte Injection, Type 1) or 0.9 % NaCl. Following randomization, hour 0 was defined as the beginning of infusion of the first bolus of blinded study treatment. Patients were assessed at baseline, hour 4 (±1), and 48 ± 6 h. The study treatment period was for up to 8 h. If the patient continued to require IVT beyond the study treatment period, the clinician ordered standard-of-care rehydration IVT. The safety follow-up period was defined from the end of the last blinded study bolus to 48 ± 6 h. The protocol recommended 20 mL/kg for the first study treatment IV bolus, but the ordered dose was left to provider discretion [21].

The primary outcome was the change in venous serum bicarbonate, as measure by total carbon dioxide by clinical chemistry automated analyzers via local laboratories, between baseline (hour 0) and hour 4. Secondary outcomes included assessments of the Gorelick score; the Baxter Animated Retching Face (BARF) [22] score for nausea/vomiting; pain (FLACC scale for ages six months to three years, FACES scale for ages 3–11 years); volume and duration of IVT; time to clinical rehydration, and length of stay in the ED. Safety assessments included physical examinations, laboratory assessments, and any reported or observed adverse events (AEs). At the safety follow-up, information was obtained regarding AEs, unplanned return visits, and hospital admission.

The intent-to-treat (ITT) population included all patients who were randomized to receive study treatment and was used to assess safety. For the primary and secondary outcomes, a modified intent-to-treat (mITT) analysis was used to focus on only those patients with a baseline serum bicarbonate level ≤22 mEq/L. This was necessary in order to allow clinicians to initiate the first study treatment bolus per standard of care prior to receipt of initial laboratory test results. However, only patients with baseline serum bicarbonate level ≤22 mEq/L continued study treatment in 10-to-20 mL/kg allotments until clinical rehydration was achieved or hour 8 transpired, whichever occurred first and was considered the “end of study” treatment. Enrolled patients with baseline bicarbonate >22 mEq/L had the study infusion stopped and were considered “early treatment release (ETR).” They did not undergo 4-h laboratory testing but were followed for safety parameters.

Sample size determination was made assuming a 25 % coefficient of variation and a 15 % difference between PLA and 0.9 % NaCl for serum bicarbonate level, following a log-normal distribution. A sample size of 80 evaluable mITT patients (40 per treatment group) had a power of 80 % to detect such a difference with a 1-sided alpha of 0.05. Planned enrollment for this study was approximately 112 subjects (56 subjects per treatment group to ensure 40 evaluable mITT subjects per treatment group), with an estimated 30 % attrition rate. The superiority of PLA in maintaining the baseline serum bicarbonate levels was established if the one-sided 95 % lower limit of the change from hour 0 to hour 4 geometric mean ratio of test/control was greater than one. Stratified analyses by age range and severity of baseline serum bicarbonate level for the primary outcome were planned a priori. For continuous efficacy and safety variables, descriptive summary statistics were provided by treatment group and time, whereas between-treatment comparisons at the post-baseline visits were performed with respect to difference or geometric mean ratio from baseline. All data are reported, but no statistical comparisons were performed for groups with <10 subjects. SAS procedures MIXED, GENMOD, and LOGISTIC (SAS OnlineDoc®, SAS Institute Inc., Cary, NC) were used to carry out all analyses. No interim analyses were planned or performed.

## Results

**Fig. 1:**
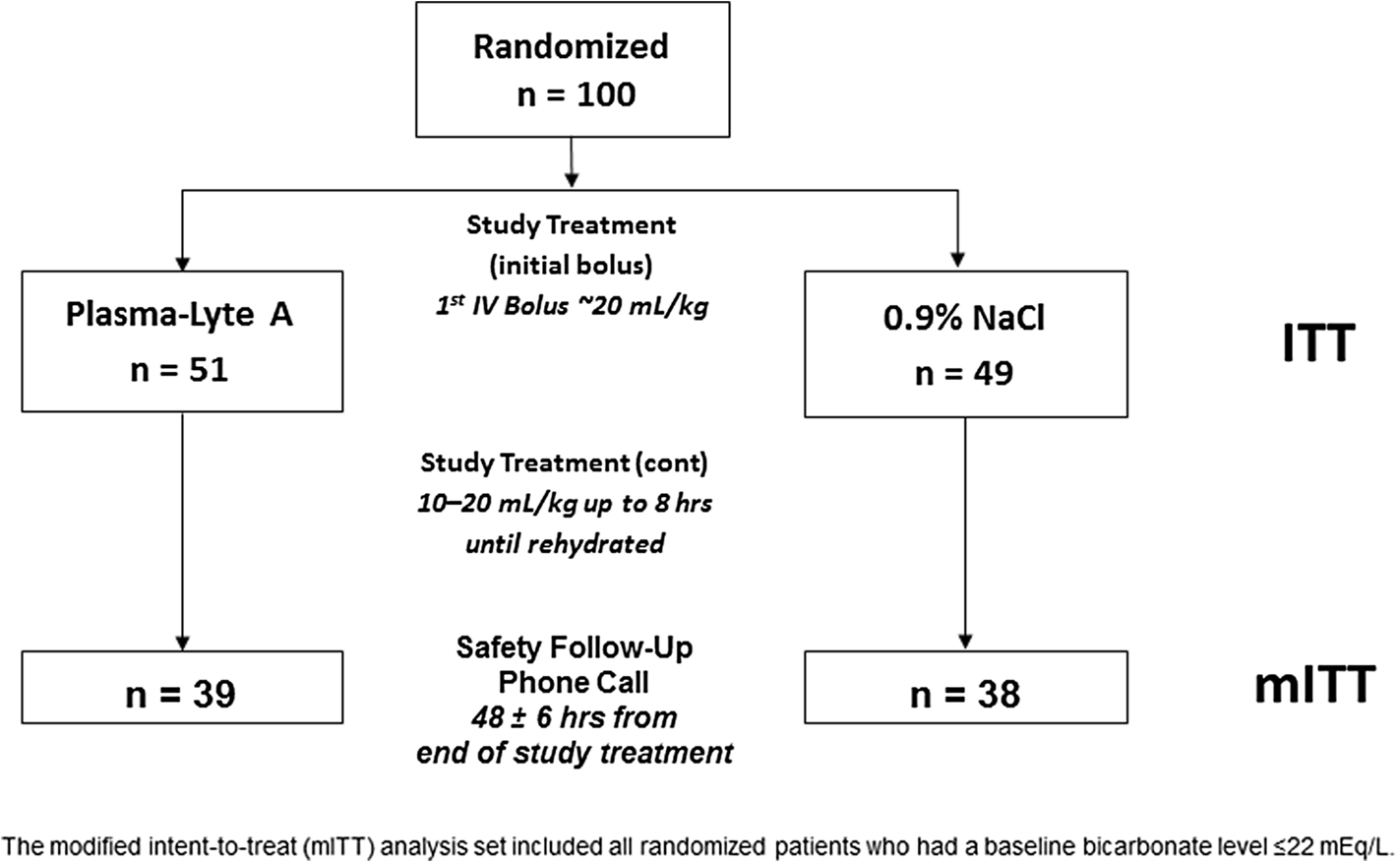
Patient disposition

Patient disposition is shown in Fig. 1. The study enrollment period was from January 20, 2011, through February 4, 2013, during which approximately 2,669 patients were prescreened for study inclusion. The exact number of patients seen is an estimate, due to different screening processes between the centers. The study was stopped early due to slow recruitment after 100 patients were enrolled with 77 evaluable mITT subjects. The ITT group was formed by 100 patients who were randomized to receive either PLA (*n* = 51) or 0.9 % NaCl (*n* = 49). ETR occurred in 23 patients due to baseline bicarbonate >22 mEq/L. There were 77 patients (PLA: 39; 0.9 % NaCl: 38) in the mITT group for assessment of the primary outcome.

### Pre-study Treatment Period Comparison

Baseline comparison of the mITT treatment groups is presented in Table 1. Of note, patients in the PLA group were older. Both groups exhibited similar Gorelick dehydration scores, as well as similar FLACC, FACES, and BARF scores. In addition, both groups had similar weight-based fluid administration prior to receipt of study fluid (PLA: 17.98 ± 11.17 ml/kg vs 0.9 % NaCl: 15.38 ± 6.55 ml/kg; Additional file 1: Table S2).

**Table 1:** Baseline clinical and biochemical characteristics (mITT population)

		Plasma-Lyte A	0.9 % NaCl
		*n* = 39	*n* = 38
Age*, months, mean		45.9	34.2
Age group, n (%)	≥6 months to ≤2 years	8 (21)	20 (53)
	>2 to ≤5 years	21 (54)	11 (29)
	>5 to <11 years	10 (26)	7 (18)
Weight*, kg		15.8 ± 5.18	13.5 ± 6.80
Vomiting episodes		8.9 ± 8.53	6.1 ± 5.92
Diarrhea episodes		4.8 ± 4.77	6.8 ± 6.77
Capillary refill time*, seconds		3.3 ± 0.50	2.8 ± 0.26
Bicarbonate, mEq/L		16.9 ± 3.51	17.8 ± 2.82
Sodium, mEq/L		137.0 ± 4.07	136.9 ± 2.93
Potassium, mEq/L		4.4 ± 0.80	4.2 ± 0.67
Chloride, mEq/L		103.0 ± 4.74	103.5 ± 4.19
BUN, mg/dL		16.5 ± 7.17	14.6 ± 6.33
Creatinine*, mg/dL		0.43 ± 0.13	0.37 ± 0.10
Glucose, mg/dL		70.3 ± 21.26	74.4 ± 21.62
Gorelick dehydration scale, median (IQR)		5 (5–6)	5 (5–6)
Ondansetron		36 (92)	29 (76)
All analgesics^a^		25 (64)	23 (61)

*BUN* blood urea nitrogen, *IQR* interquartile range

All data are presented as mean ± standard deviation unless otherwise indicated

^a^Acetaminophen, nonsteroidal anti-inflammatory drugs, and narcotics administered orally and/or by intravenous infusion

*P <0.05 PLA vs 0.9 % NaCl

### Treatment and outcomes

There were no significant differences in volume, duration of fluid administration, or maintenance IVT between groups. The number of boluses was 1.9 for both groups, dose of study treatment was 38.4 mL/kg vs 39.6 mL/kg, duration of administration was 104.3 min vs 93.7 min and maintenance fluid was 12.2 vs 12.3 mL/kg PLA vs 0.9 % NaCl respectively, all *P* > .05. Twelve patients in the PLA group received concomitant maintenance IV fluid vs 11 in the 0.9 % NaCl group.

Outcome comparisons between the two groups are presented in Table 2. Patients receiving PLA demonstrated significantly greater increase in serum bicarbonate levels from baseline to hour 4 compared with patients receiving 0.9 % NaCl. When stratified by bicarbonate level severity, PLA showed superiority over 0.9 % NaCl treatment for bicarbonate ranges ≥12 to 16. Although PLA patients in the >16 to 22 mEq/L bicarbonate range had a statistically significant increase in serum bicarbonate levels (*P* < .05), the increase in the PLA group was not significantly different from the 0.9 % NaCl group (*P* = .11) for this bicarbonate range. None of the patients receiving 0.9 % NaCl had baseline serum bicarbonate level <12 mEq/L. The three patients with severe acidosis who received PLA demonstrated a mean improvement in serum bicarbonate level from 9.3 ± 0.6 mEq/L to 14.3 ± 4.2 mEq/L.

**Table 2:** Primary and secondary outcomes (mITT population)

	Plasma-Lyte A	0.9 % NaCl	*P* value
	*n* = 39	*n* = 38	
Bicarbonate <23 mEq/L			
Baseline (hour 0)	16.9 ± 3.51	17.8 ± 2.82	.004
Hour 4	18.5 ± 3.74	18.0 ± 3.67	
Bicarbonate <12 mEq/L			
Baseline (hour 0) (n)	9.3 ± 0.58 (3)	– (0)	NA^b^
Hour 4 (n)	14.3 ± 4.16 (3)	– (0)	
Bicarbonate ≥12–16 mEq/L			
Baseline (hour 0) (n)	14.5 ± 1.34 (13)	14.6 ± 1.29 (13)	.04
Hour 4 (n)	16.1 ± 2.28 (12)	14.7 ± 2.90 (11)	
Bicarbonate >16–22 mEq/L			
Baseline (hour 0) (n)	19.23 ± 1.86 (23)	19.51 ± 1.68 (25)	.11
Hour 4 (n)	20.35 ± 3.18 (22)	19.53 ± 2.95 (24)	
Chloride, mmol/L			
Baseline	103.03 ± 4.74	103.53 ± 4.19	<0.001
Hour 4	104.49 ± 3.18	108.51 ± 4.87	
Gorelick dehydration scale			
Baseline (hour 0)	5.2 ± 0.93	5.3 ± 1.11	.03
Hour 2	2.0 ± 1.45	2.8 ± 1.74	
Hour 4	0.81 ± 0.84	1.41 ± 1.08	.08
FLACC pain scale			
Baseline (Hour 0)	2.0 ± 1.91	1.7 ± 2.00	.03
Hour 2	0.6 ± 0.98	1.7 ± 2.59	
Hour 4	1.44 ± 2.18	0.68 ± 1.35	.15
FACES pain scale			
Baseline (Hour 0)	2.3 ± 1.86	3.2 ± 1.90	.31
Hour 2	1.1 ± 1.55	1.9 ± 1.60	
Hour 4	0.37 ± 0.60	1.11 ± 1.54	NA
BARF Scale			
Baseline (Hour 0)	5.10 ± 4.02	4.43 ± 3.86	.27
Hour 2	1.5 ± 3.10	2.00 ± 2.58	.55
Hour 4	0.3 ± 0.98	1.23 ± 2.24	.12
Time to Rehydration^a^, h	6.1 ± 1.75	7.0 ± 2.7	.13
Hospitalized, n (%)	12 (31)	11 (29)	.86

*BARF*, Baxter animated retching faces; *FLACC*, face, legs, activity, cry, consolability pain assessment scale for children

All data are presented as mean ± standard deviation unless otherwise indicated

^a^Rehydration was defined as the time the clinician determined no further bolus fluid therapy was indicated

^b^Not available due to sample size (n <10)

Comparison by age strata demonstrated PLA superiority for the >2 years to ≤5 years age range (mean 17.16 to 18.31; *P* < .05). Although the >5 years to <11 years age range had a significant increase in serum bicarbonate level for PLA patients (*P* < .05), superiority over 0.9 % NaCl was not calculated due to the small number of older subjects in the 0.9 % NaCl group. Patients receiving PLA demonstrated significant improvements in clinical status as measured by the hour 2 Gorelick score and FLACC pain scales. Patients in the PLA group had improvement in BARF scores from baseline to hour 2 (mean change −3.6 ± 5.1; *P* = .005) and hour 4 (mean change −4.8 ± 3.97; *P* = .005). Patients in the 0.9 % NaCl group did not show similar BARF score improvements at hour 2 (mean change−2.1 ± 4.0; *P* = .08) but did at hour 4 (mean change −2.9 ± 3.88; *P* = .02). However, between-group BARF scores were not significant. No other differences in secondary outcome measures were observed.

### Safety outcomes

**Fig. 2:**
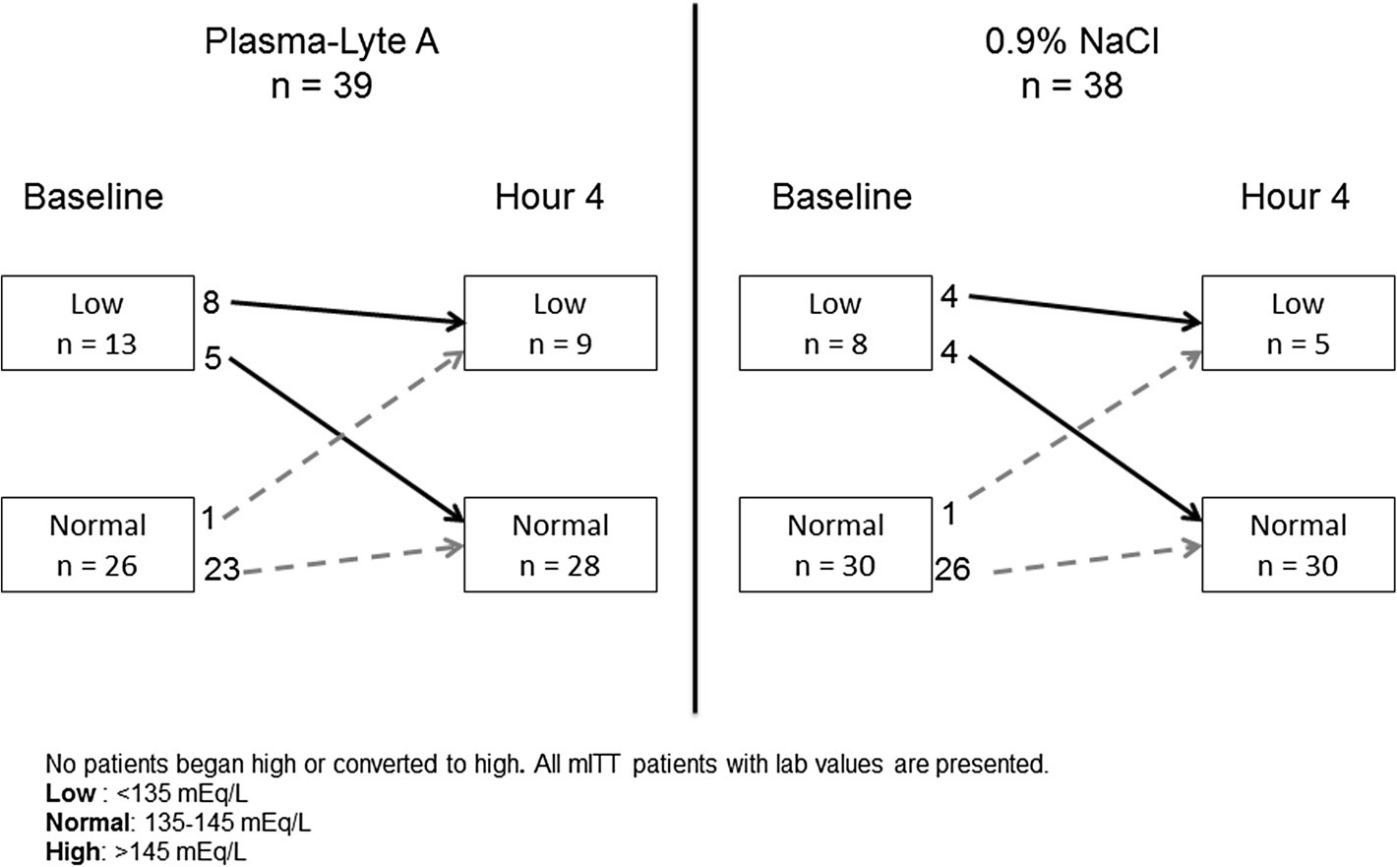
Change in sodium levels from baseline to hour 4

**Fig. 3:**
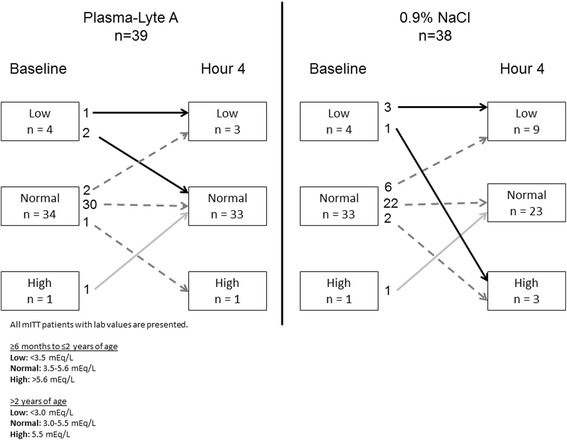
Change in potassium levels from baseline to hour 4

The shift in potassium and sodium levels from baseline to hour 4 is presented in Figs. 2 and 3. There were no episodes of hyponatremia (<130 mEq/L) or hypernatremia (>155 mEq/L) in either group. Some patients in each group presented with hyponatremia that was also documented at hour 4 (PLA: 8/13; 0.9 % NaCl: 4/8), and one patient in each group developed mild hyponatremia (131–135 mEq/L). Hypokalemia (<3.5 mEq/L for ages ≥6 months to ≤2 years and <3.0 mEq/L for ages 2–11 years) and hyperkalemia (>5.6 mEq/L for ages ≥6 months to ≤2 years and >5.5 mEq/L for ages 2–11 years) were both assessed. Two patients in the PLA group vs 6 in the 0.9 % NaCl group became hypokalemic at hour 4. Four patients in the study developed hyperkalemia (PLA: 1/39 vs 0.9 % NaCl: 3/38) by hour 4. All samples showing hyperkalemia demonstrated hemolysis, all of which were deemed clinically insignificant by the investigators.

## Discussion

This prospective, randomized, triple-blind, multicenter trial comparing PLA to 0.9 % NaCl in children with dehydration secondary to AGE determined that PLA was superior to 0.9 % NaCl for improving the metabolic acidosis (change from baseline in bicarbonate of 1.6 mEq/L for PLA vs 0.0 mEq/L for 0.9 % NaCl) despite comparable initial serum bicarbonate levels and comparable volumes of IVT. In addition to acidosis, pain as assessed by the FLACC scale, and dehydration score significantly improved at hour 2 in patients receiving PLA. The clinical significance of the improved dehydration scores at hour 2 is unclear as the dehydration scores were not significantly improved at hour 4 in patients receiving PLA. It may be that once the intravascular fluids redistributed, the clinical picture was similar between the two treatment groups. Importantly, neither PLA nor 0.9 % NaCl induced significant hyponatremia or hyperkalemia. In fact, the frequency of conversion to abnormal laboratory values was similar between groups.

Although randomization was performed at the center level, the distribution of the children’s age and weight was dissimilar between groups, precluding some test comparisons due to small sample size. However, there were no differences in weight-based rehydration volumes or time despite baseline differences in age and capillary refill. Age stratification demonstrated a similar treatment effect across age groups.

We tested serum bicarbonate as the primary outcome of this study based on evidence suggesting that dehydration severity is related to bicarbonate [23]. Low serum bicarbonate levels are the most common electrolyte abnormality occurring in children with AGE, and dehydration severity has been found to be related to the bicarbonate concentration on admission [24, 25]. Bicarbonate is also the electrolyte most likely to alter care [25] in that it correlates with extended stay, admission to the observation unit, and administration of more IVT. Bicarbonate is a quantifiable, objective determinant, thus it is ideal for statistical power determination [9]. The European Society for Paediatric Gastroenterology, Hepatology, and Nutrition/European Society for Paediatric Infectious Diseases recommends that serum bicarbonate laboratory tests be considered in dehydrated children if IVT is started [26]. These guidelines also recommend rapid rehydration with 20 mL/kg/h of 0.9 % saline solution for 2 to 4 h.

The high level of chloride in 0.9 % NaCl is known to be associated with hyperchloremic metabolic acidosis. Therefore, it is not surprising that despite its ability to rehydrate in this study, 0.9 % NaCl did not improve metabolic acidosis. Our study determined that correcting acidosis and fluid status was associated with clinically relevant outcomes of significant improvement in dehydration scores and abdominal pain. In addition, although the study was not powered to detect a difference in time to rehydration, the data suggest that there may be a clinically meaningful difference of nearly 1 h (*P* = .13) and may warrant further study with a larger sample.

Interestingly, 0.9 % NaCl induced hyperchloremia without an associated change in bicarbonate. This finding is in contrast to studies in children with severe AGE [12] where it increased bicarbonate, and in contrast to adult studies that show when 0.9 % NaCl is used as a resuscitative fluid, it generally decreases serum bicarbonate [27–29].

When 0.9 % NaCl is used to treat children with acute diarrhea and severe dehydration, pH may decrease despite improvement in clinical signs of rehydration [30] In the present study the finding that 0.9 % NaCl caused a significant increase in serum chloride levels suggests that 0.9 % NaCl may exacerbate AGE acidosis via an ensuing non-anion gap metabolic acidosis, due to a rapid rise in serum chloride levels relative to sodium [29].

The majority of children were rehydrated with approximately 40 mL/kg of treatment fluid. Although we allowed treatment for up to 8 h, most were rehydrated with bolus infusions of less than 2 h and independent of IVT type, approximately one-third in each group needed observation/inpatient admission for continued hydration.

We also found that electrolyte abnormalities post-treatment were similar between the two treatment groups, alleviating concerns of using a solution that contains a lower sodium concentration than 0.9 % NaCl and that contains potassium.

While common practice does not introduce potassium until urine output is observed, this study supports the safe use of a fluid containing physiologic amounts of potassium. In fact, the amount of potassium delivered by PLA treatment during the study is surprisingly small–PLA contains 5 mEq/L of potassium. The average child in the PLA group weighed approximately 16 kg. At 40 mL/kg, the average child received a total of 3.2 mEq of potassium via PLA, which is equivalent to about one-third of a banana.

The strengths of this study were its randomization, effective blinding, clinical equipoise, and multicenter design. Limitations were its relatively small sample size and the resultant inability to detect some clinically important secondary outcomes. Although the primary objective was achieved despite a lower-than-planned enrollment, not meeting planned enrollment may have impaired our ability to detect some of the secondary outcomes. Moreover, although the random assignment was not similar in terms of age or weight, the weight-based volumes delivered were similar. The planned evaluation of the impact of PLA by severity of baseline acidosis as measured by the bicarbonate strata was limited by the lack of severely dehydrated children in the 0.9 % NaCl group.

## Conclusion

This study adds new information for the treatment of AGE-induced dehydration. PLA is an appropriate alternative to 0.9 % NaCl because it provides the necessary water and electrolyte replacement, and as an alkalinizing agent it may ameliorate the clinical sequelae of AGE-induced acidosis. Both PLA and 0.9 % NaCl were effective and well tolerated and had similar safety profiles. Plasma-Lyte A was more effective than 0.9 % NaCl at correcting acidosis, in particular in patients with moderate acidosis on admission, and led to improved clinical findings of dehydration.

## Abbreviations

AGE, Acute gastroenteritis; BARF, Baxter Animated Retching Face; ED, Emergency department; FLACC, Face, legs, activity, cry, consolability pain assessment scale for children; HCA, Hyperchloremic metabolic acidosis; IVT, Intravenous fluid therapy; LR, Lactated Ringer’s; mITT, Modified intent-to-treat; NaCl, Sodium chloride; PLA, Plasma-Lyte A (sodium chloride, sodium gluconate, sodium acetate, potassium chloride and magnesium chloride

## Additional file

Additional file 1: Table S1. Ethics Committees. **Table S2.** IVF bolus during screening and safety follow-up visits (mITT population). (DOCX 15 kb)
