# Supplementary material for: A randomized trial of Plasma-Lyte A and 0.9 % sodium chloride in acute pediatric gastroenteritis
Source: BMC Pediatr. 2016 Aug 2;16:117. doi: 10.1186/s12887-016-0652-4 (PMC4969635; doi:10.1186/s12887-016-0652-4)
Supplement: Additional file 1: Table S1. — Ethics Committees. Table S2. IVF bolus during screening and safety follow-up visits (mITT population). (DOCX 15 kb) [file 12887_2016_652_MOESM1_ESM.docx]

**Additional file 1**

**Table S1**. Ethics Committees

| **United States** |  |
| --- | --- |
| Emory University/Children’s Healthcare of Atlanta |  |
| Cincinnati Children’s Hospital Medical Center |  |
| Cleveland Clinic Institutional Review Board |  |
| Oregon Health and Science University |  |
| Seton Institutional Review Board |  |
| Children’s Hospital of Wisconsin |  |
| **Canada** |  |
| University of Calgary |  |
| The University of British Columbia / Children’s and Women’s Health Centre of British Columbia Research Ethics Board |  |

**Table S2.** IVF bolus during screening and safety follow-up visits (mITT population)

|  | **Plasma-Lyte A n = 39** | **0.9% NaCl n = 38** |
| --- | --- | --- |
| **Prior to Screening** | | |
| **All IV fluids (0.9% NaCl)** |  |  |
| Mean±SD total volume*, mL/kg | 17.98±11.17 (n=17) | 15.38±6.55 (n=13) |
| Mean±SD duration*, min | 48.82±36.44 (n=17) | 44.17±14.75 (n=12) |
| **Safety follow-up** | | |
| **All IV fluids**^a^ |  |  |
| Mean±SD total volume, mL/kg | 17.34±12.44 (n=5) | 33.87±18.21 (n=7) |
| Mean±SD duration, min | 75.00±42.24 (n=5) | 227.50±193.18 (n=6) |
| **0.9% NaCl**^a^ |  |  |
| Mean±SD total volume, mL/kg | 25.08±9.22 (n=3) | 35.18±13.65 (n=3) |
| Mean±SD duration, min | 59.67±39.12 (n=3) | 112.50±31.82 (n=2) |
| **D5 0.45% NaCl**^a^ |  |  |
| Mean±SD total volume, mL/kg | 5.73±0.65 (n=2) | 20.92±11.68 (n=2) |
| Mean±SD duration, min | 98.00±48.08 (n=2) | 317.00±233.35 (n=2) |
| **D5 0.9% NaCl**^a^ |  |  |
| Total volume, mL/kg | — | 66.04 (n=1) |
| Duration, min | — | 462 (n=1) |
| **Lactated Ringer’s**^a^ |  |  |
| Total volume, mL/kg | — | 23.62 (n=1) |
| Duration, min | — | 44.00 (n=1) |

*P>0.05 based on Wilcoxon 2 sample test

^a^Statistical test not available due to sample size (n <10).
